# Preparation of Crystallites for Oriented Poly(Lactic Acid) Films Using a Casting Method under a Magnetic Field

**DOI:** 10.3390/polym10101083

**Published:** 2018-09-29

**Authors:** Shuta Hara, Shuto Watanabe, Kohki Takahashi, Shigeru Shimizu, Hiroki Ikake

**Affiliations:** 1 Department of Materials and Applied Chemistry, College of Science and Technology, Nihon University, 1-8-14 Kandasurugadai, Chiyoda-ku, Tokyo 101-8308, Japan; hara.shuta@nihon-u.ac.jp (S.H.); shuto.watanabe@polymer.chem.cst.nihon-u.ac.jp (S.W.); shimizu.shigeru@nihon-u.ac.jp (S.S.); 2 Institute for Materials Research, Tohoku University, 2-1-1 Katahira, Aoba-ku, Sendai, Miyagi 980-8577, Japan; kohki@imr.tohoku.ac.jp

**Keywords:** poly-l-lactic acid, magnetic orientation, ionic liquid, α-crystal

## Abstract

Poly-l-lactic acid (PLLA) has biocompatibility and unique characteristics such as piezoelectric properties. This attracts attention not only in the environmental field but also in the biomedical and electronic materials fields. In recent years, the literature about orienting PLLA crystals has been promoting new applications for PLLA such as high strength fiberization and piezoelectric properties. This paper presents a new technique to orient the PLLA crystalline through casting under magnetic irradiation. The advantage of this technique is that it is possible to radiate the magnetic field to the PLLA crystalline in an extremely low viscosity environment. Moreover, the heat treatment condition was optimized in order to improve the low crystallinity of casting, and it succeeded in producing a PLLA film with a high degree of orientation and high crystallinity. Furthermore, PLLA/1-butyl-3-methylimidazolium dibutylphosphate (bmimjdbp) composite films were prepared under the same conditions, and this also succeeded in the further improvement of crystallinity.

## 1. Introduction

In recent years, poly(l-lactic acid) (PLLA) has been well-known as a novel material which can be used to save petroleum resources and reduce carbon dioxide. PLLA has also attracted increasing attention in ecological fields, in the biomedical field [1,2], and in electronic devices [3] because of its unique properties, such as its nontoxicity to the human body and the piezoelectric effect [2,4].

Much effort has been made to regulate the PLLA crystal due to how greatly this influences its macro-physical properties. Depending on the crystallization conditions, PLLA exhibits different crystalline forms, such as alpha-, beta-, and gamma-forms and their mixtures [5,6,7,8]. In general, the alpha-crystal of PLLA is usually prepared under ordinary conditions, such as through melting or solutions. It is orthorhombic and has a 10_3_ helical chain conformation [5].

To study the crystal form and crystal orientation of PLLA for new applied research, such as high strength fiberization and the impartation of piezoelectric properties, it has been reported that the stretch orientation method has a high reliability as a method of orienting crystals of PLLA. In using this method, however, there was an issue that the piezoelectric characteristics, mechanical properties, and reduction of the melting temperature of the crystal were deteriorated because a part of the α-crystal changed to β-crystal during the operation process. This indicates that the α-form crystal structure, which has a left-handed 10_3_ helical conformation, was forcibly stretched to change into a β-form crystal structure, which has a three-fold helical conformation [5,6].

In previous works, we have reported that the magnetic-induced blend films consisting of PLLA and poly(dl-lactic acid) (PDLLA) were prepared using an isothermal process under a magnetic-field of 10 T without the formation of β-crystals [9], and there was an improved degree of crystallization maintaining its degree of orientation [10]. In this method, it was necessary for a higher magnetic torque to be transmitted than the resistance force due to a molten viscosity of PLLA. Accordingly, PLLA films had higher crystallinity and but lower orientation.

In this study, we focused on the crystallization of PLLA using the cast method under a magnetic field because of the low viscous environment this provided during crystallization, crystal growth, and the orientation process. Furthermore, we added 1-butyl-3-methylimidazolium dibutyl phosphate ([bmim]dbp), which is an ionic liquid (IL), to PLLA in order to improve the isothermal crystallization rate of PLLA [11]. We prepared the oriented PLLAIL composite film under a magnetic field.

## 2. Experimental Method

### 2.1. Materials

Ethyl acetate, methanol, chloroform, toluene, and *n*-hexane were purchased from Kanto Chemical Co., Tokyo, Japan. Tin(II) octylate, 1-methylimidazole, and tributyl phosphate were obtained from Tokyo Chemical Industry Co., Tokyo, Japan. l-lactide was purchased from Musashino Chemical Laboratory Ltd, Tokyo, Japan.

### 2.2. Synthesis of PLLA

PLLA was synthesized using the following procedure. l-lactide of 100 g was dissolved in 120 mL of ethyl acetate with heating at 70 °C and recrystallized by being incubated in ice for 3 h. Purified solid l-lactide monomer of 30 g and 0.5 M tin(II) octylate toluene solution of 104 μL were put into a glass tube and that was sealed [12,13]. Then, the glass tube was continuously heated at 150 °C for 0.5 h, 130 °C for 5 h, 110 °C for 13 h, and 90 °C for 5 h in the oil bath. The obtained PLLA was a yellowish white solid before it was dissolved in 400 mL chloroform. Finally, the solution was added dropwise to 1.5 L of methanol, and PLLA was obtained as a white precipitate. The ^1^H NMR (400 MHz, Chloroform-d, 25) spectra of PLLA exhibits the signal of the methine group at 5.16 ppm (dd, 1H) and the signal of the methyl group at 1.58 ppm (d, 3H).

### 2.3. Synthesis of [bmini]dbp

[bmim]dbp were prepared according to the method described by Nie et al. [14,15]. Tributyl phosphate (66.6 mmol, 17.74 g) and 1-methylimidazole (66.6 mmol, 5.46 g) were mixed in a three-necked flask (200 mL). The mixture was heated at 150 °C for 24 h in a nitrogen atmosphere. The reaction mixture was washed several times with *n*-hexane, and the solvent was removed by vacuum drying to obtain the desired product in the form of a brownish liquid. The chemical shifts of the 400 MHz ^1^H NMR spectra of [bmim]dbp in chloroform-d at 25 °C were recorded as follows: $\delta_{H}=$ 10.26 (s, 1H), 7.61 (t, 1H), 7.46 (t, 1H), 4.28 (t, 2H), 4.00 (s, 3H), 3.86 (dd, 4H), 1.87 (m, 2H), 1.59 (m, 4H), 1.37 (m, 6H), and 0.92 (m, 9H).

### 2.4. Preparation of Oriented PLLA Films 

The oriented PLLA film was prepared using the solution casting method under a magnetic field. PLLA of 0.2 g was dissolved in 12 mL of chloroform, and the solution was poured in a glass dish with a diameter of 4 cm. The glass dish was placed in the magnetic flux density of 10 T by using a superconducting magnet (10T100-CSM, Institute for Materials Research, Tohoku University) at room temperature until the solvent volatilized.

Oriented PLLA containing 20 wt% of IL film was prepared using the same procedure. PLLA with 0.2 and 0.04 g of IL dissolved in 12 mL of chloroform and was poured in a petri dish and was placed in a 10 T magnetic field at room temperature. Each control samples was prepared under similar conditions without the applied magnetic field.

## 3. Characterization 

Molecular weight was measured using the viscosity method in a dilute PLLA chloroform solution with an Ubbelohde capillary viscometer (Shibayama Scientific Co., Ltd., Tokyo, Japan) at 25 °C. The viscosity average molecular weight, $M_{v}$, was used to calculate the intrinsic viscosity, [η], by using following equation [16],

(1) $$\left[\eta \right]=5.45\times 10^{-4}M_{v}^{0.73}$$

The calculated $M_{v}$ of PLLA was $5.2\times 10^{4}$. The optical purity measurement of PLLA was carried out with a sodium lamp (λ = 589 nm) and an optical path length of 10 cm using the P-2300 optical rotation meter (JASCO Corporation, Tokyo, Japan). The optical purity of PLLA was 97.4%. Wide angle X-ray diffraction (XRD) measurement was carried out using a X’Pert PRO MPD (PANalytical, Tokyo, Japan) operated at 45 kV and 40 mA to generate a Ni-filtered Cu-Kα X-ray beam. The scanning speed was 0.01°/s, and the measurement range was 3–60° at room temperature. The size of the lamellar crystal was calculated from the full width at the half maximum (FWHM) of the highest intensity diffraction peak, which is based on the Debye–Scherrer equation,

(2) $$t=\frac{0.9\lambda }{b\cos\theta }$$

where t is the crystallite size, λ is the wavelength of Cu-Kα radiation, b is the FWHM, and θ is the diffraction angle of the strongest peak. Crystal growth in the films was observed using a polarization microscope (BX53-33P-OC-1, Olympus, Tokyo, Japan).

## 4. Results and Discussion

### 4.1. Characterization of Oriented PLLA Film 

**Figure 1:**
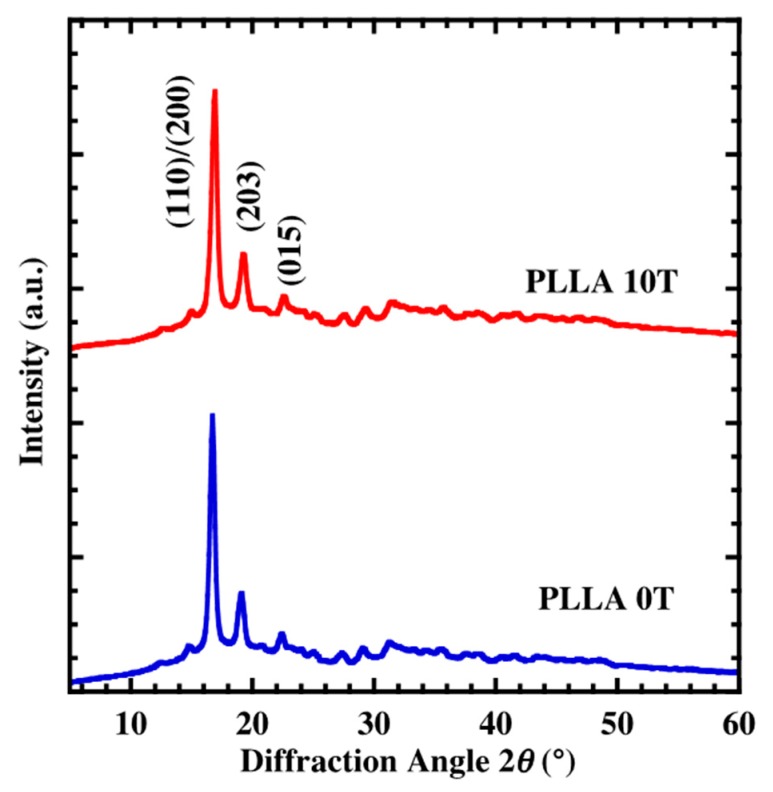
XRD patterns of PLLA10T and PLLA0T films.

**Figure 2:**
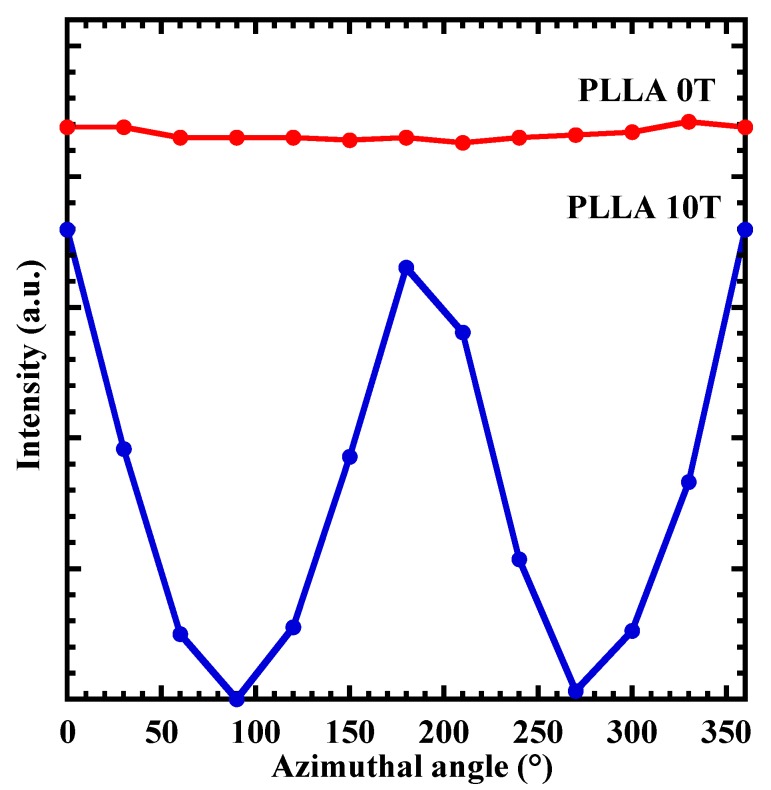
XRD (110)/(200)intensity along the azimuthal angle for PLLA0T and PLLA10T.

The XRD pattern of neat PLLA cast film (PLLA0T) and oriented film (PLLA10T) is shown in Figure 1. Both films showed diffraction peaks at 2θ = 16.8, 19.2, and 21.5°, which can be assigned to the (110)/(200), (203), and (015) planes [17], respectively. These diffraction peak positions correspond to that of the α-form crystal. This characteristic of the XRD pattern is the same as those of the α-form crystal, indicating that only the α-form PLLA crystal was produced under the conditions we used. Figure 2 shows the result of X-ray azimuthal scans along the (110)/(200) plane of PLLA0T and PLL10T. It is clear that PLLA10T exhibits azimuthal angular dependence. The degree of orientation was defined as the azimuthal angle of the diffracted intensity of (110)/(200) planes. The degree of the magnetic-field-induced orientation, $f_{c}$, was calculated with Herman’s orientation factor [18], as defined by the following equations

(3) $$f_{c}=\frac{3<\cos^{2}\phi >-1}{2}$$

(4) $$\;<\cos^{2}\phi >=\frac{\int_{0}^{\frac{\pi }{2}}I\left(\phi \right)\cos^{2}\phi \sin\phi d\phi }{\int_{0}^{\frac{\pi }{2}}I\left(\phi \right)\sin\phi d\phi }\;$$

where ϕ is the azimuthal angle and I(ϕ) is the azimuthal intensity. The calculated $f_{c}$ of PLLA0T and PLLA10T were 15 and 78%, respectively. The $f_{c}$ of PLLA10T, 78%, was larger than that found using the thermal melting method [9,10]. These results suggested that the lamellar crystal of PLLA created using the casting method was oriented in a lower viscosity environment.

### 4.2. Crystal Growth of Oriented Film 

**Figure 3:**
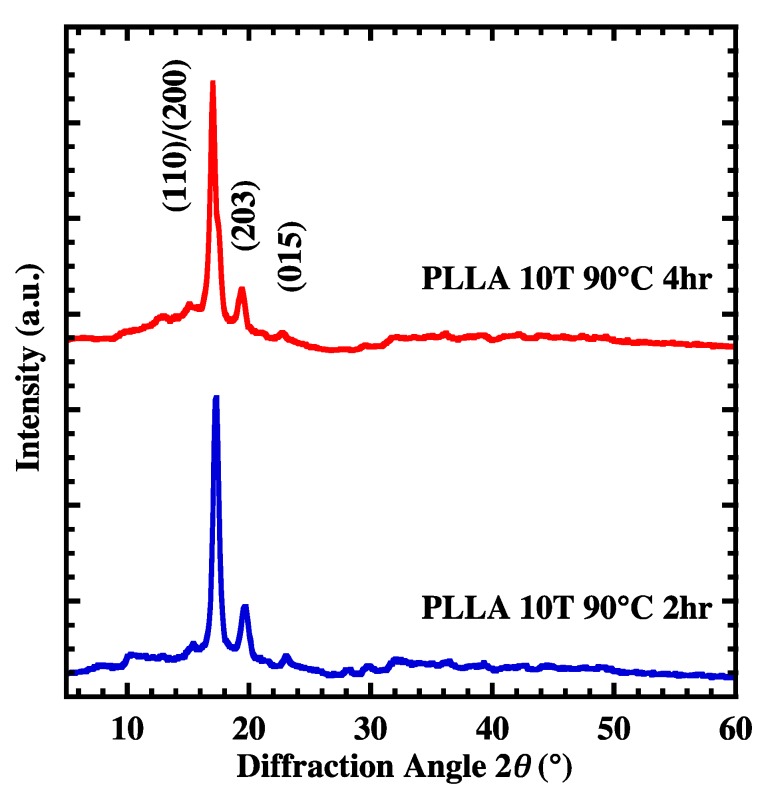
XRD patterns of PLLA10T2 and PLLA10T4 films.

In Figure 3, XRD patterns for PLLA10T, which were heated at 90 °C for 2 h (PLLA10T2) and 4 h (PLLA10T4), were shown. Both films show two dominant diffraction peaks at 2θ=16.8 and 19.2° which represent the (200)/(100) and (203) planes, respectively, and the weak diffraction peak at 21.5° is indicative of the (015) plane. The degree of crystallization, $X_{c}$, of PLLA was evaluated from the peak areas of the XRD pattern using following the equation.

(5) $$X_{c}\%=\frac{100S_{c}}{S_{c}+S_{a}}$$

where $S_{c}$ and $S_{a}$ are crystalline and amorphous diffraction areas, respectively. $X_{c}$ and the lamellar crystal size (t) of PLLA10T, PLLA10T2, and PLLA10T4 were summarized in Table 1. $X_{c}$ increased with increasing annealing time, then reached a constant value at 4 h. The annealing time dependence of $X_{c}$ for PLLA0T film was also evident (Figure S1). The crystal size calculated from Equation (2) increased with increasing annealing time for all samples. This shows that, irrespective of the degree of crystal orientation, crystal growth proceeds for at least 4 h as annealing time is increased.

**Figure 4:**
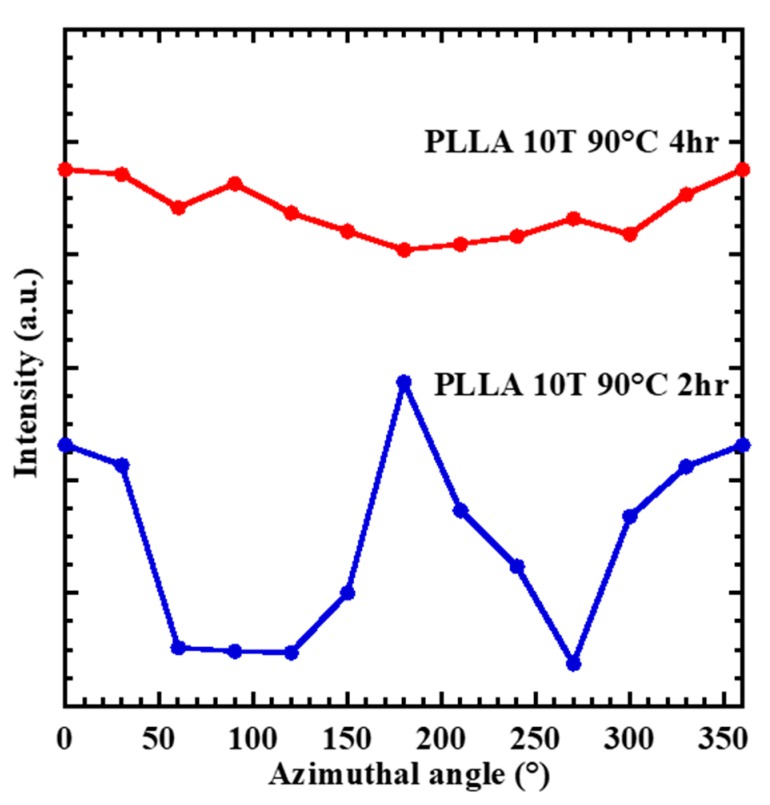
XRD (110)/(200) intensity along the azimuthal angle for PLLA10T2 and PLLA10T4 films.

**Figure 5:**
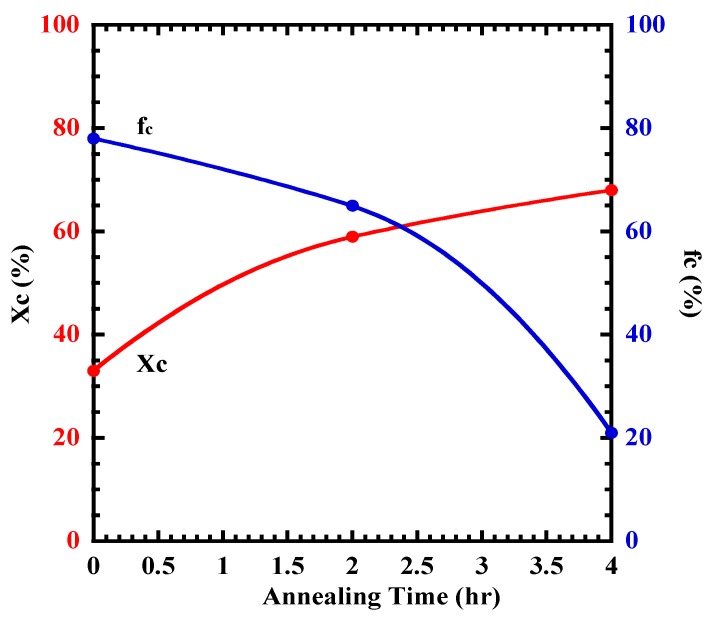
Relationship between *X*_c_ and $f_{c}$ during the heat treatment of oriented PLLA film.

While PLLA10T2 shows azimuthal angle dependence, PLLA10T4 is independent of the azimuthal angle. That is, the $f_{c}$ of PLLA10T2 is larger than that of PLLA10T4 (Figure 4). Figure 5 shows the relationships between $X_{c}$ and annealing time and between $f_{c}$ and annealing time. With increasing annealing time, $X_{c}$ monotonically increases, whereas $f_{c}$ monotonically decreases. This means that the annealing process could contribute to crystal growth but not to orientation.

### 4.3. Crystal Growth and Orientation of [bmim]dbp Composite PLLA

**Figure 6:**
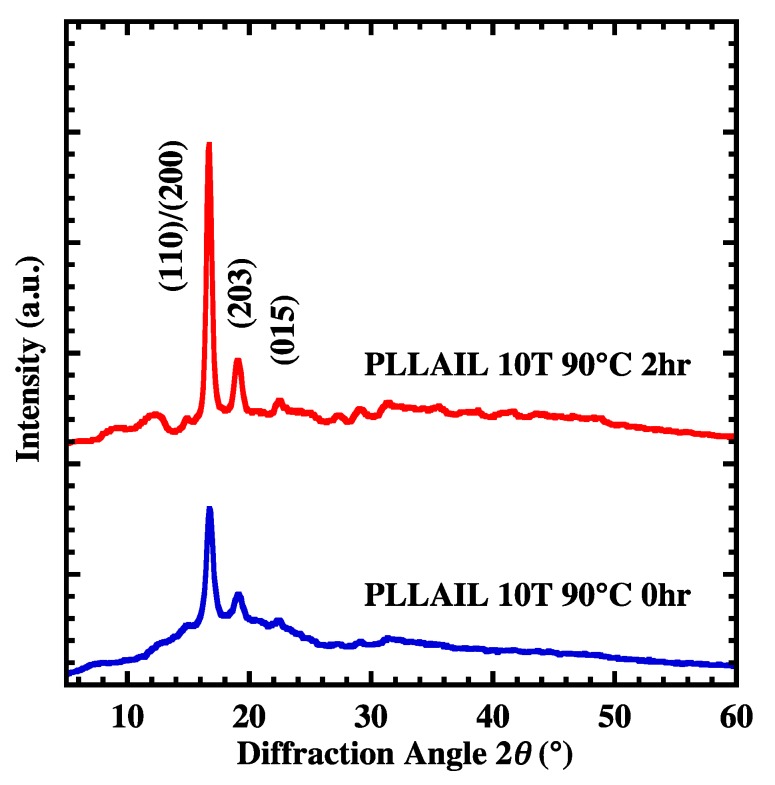
XRD patterns of PLLAIL10T and PLLAIL10T2 films.

**Figure 7:**
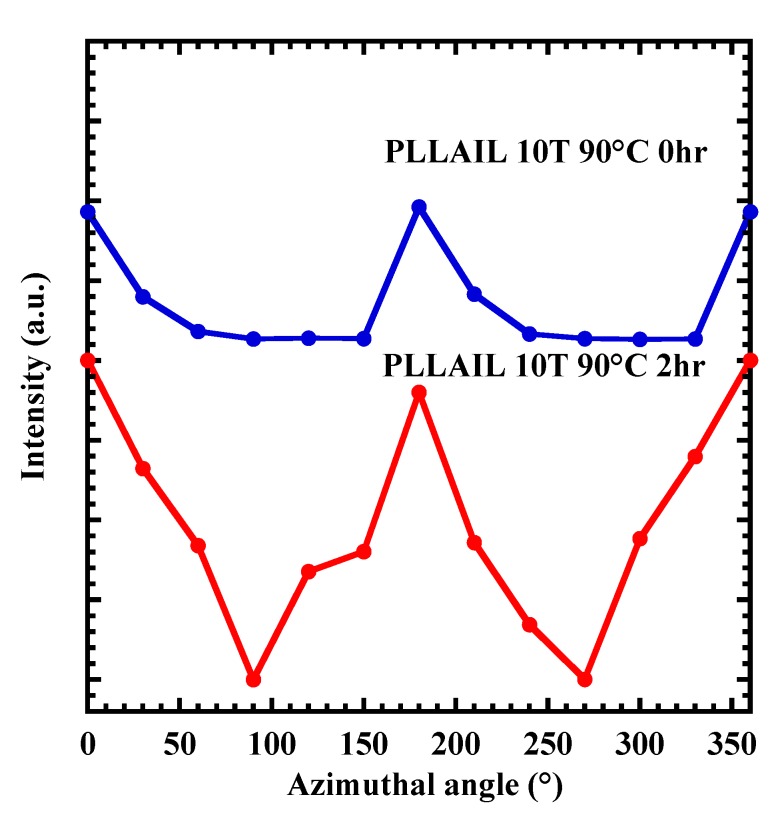
XRD (110)/(200) intensity along the azimuthal angle for PLLAIL10T and PLLAIL10T2 films.

The annealing time dependence of $X_{c}$ for PLLAIL is shown in Figure S2. $X_{c}$ increases with increasing annealing time. This behavior is similar to what was found during PLLA crystallization. Figure S3 also illustrated the XRD pattern for PLLAIL. Since XRD patterns were not influenced by the addition of IL, the crystal form of all samples was assigned to an α-form crystal based on diffraction peaks. Both PLLAIL10T and PLLAIL10T2, based on the results of XRD measurement, have an α-crystal form, and their $X_{c}$ was 28 and 65%, respectively (Figure 6). In Figure 7, the azimuthal angle dependence of PLLAIL10T and PLLAIL10T is shown. The degree of orientation $f_{c}$ was calculated using Equations (3) and (4). The values of $f_{c}$ were 82% for PLLAIL10T and 62% for PLLAIL10T2. A small decrease of $f_{c}$ for PLLAIL10T2 might be explained by the activation of thermal fluctuation from the addition of [bmim]dbp.

### 4.4. Changing the Crystal Morphology of Oriented PLLA and PLLAIL with Heating Treatment

**Figure 8:**
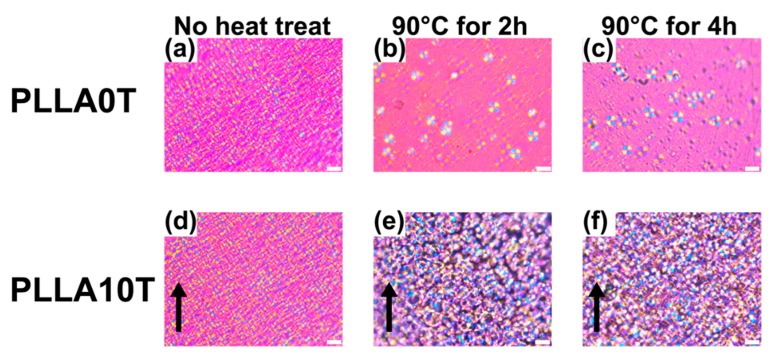
Polarization microscope images of PLLA films: (**a**) PLLA0T, (**b**) PLLA0T2, (**c**) PLLA0T4, (**d**) PLLA10T, (**e**) PLLA10T2, and (**f**) PLLA10T4. The black arrow indicates the direction of the magnetic field. White bar = 50 μm.

Figure 8 shows a polarizing optical microscopy (POM) image of PLLA0T film and PLLA10T films with an annealing time from 0 to 4 h at 90 °C. In PLLA0T, crystal growth was confirmed by increasing heating time. PLLA0T film shows the formation of spherulite, and its content increases with increasing annealing time. That is, $X_{c}$ increases with increasing annealing time. This result corresponds to the result of the $X_{c}$ of PLLA0T estimated from XRD. The increasing spherulite radius of PLLA0T heated at 90 °C is in agreement with the data obtained by Tsuji and Ikada [19].

**Figure 9:**
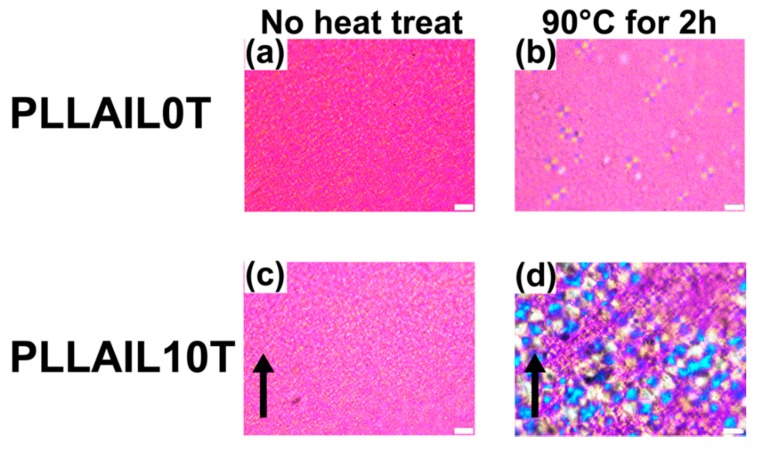
Polarization microscope images of PLLA films: (**a**) PLLAIL0T, (**b**) PLLAIL0T2, (**c**) PLLAIL10T, and (**d**) PLLAIL10T2. The black arrow indicates the direction of the magnetic field. White bar = 50 μm.

On the other hand, in PLLA10T, there was increased crystal growth with increased annealing time, but there were no observed spherulites. A similar phenomenon was observed for PLLAIL10T, and the lamella crystal size of PLLAIL10T2 was larger than that of PLLA10T2 (Figure 9). Moreover, these morphologies were isotropic independent of the irradiation direction of the magnetic field. However, taking XRD analysis of oriented lamellar crystals into account, it can be inferred that the oriented lamellar crystals do not organize into spherulite because the growth of spherulite requires isotropic lamellar crystals. These results supported that the lamellar crystals are oriented.

## 5. Conclusions

The oriented PLLA film prepared using the combining casting method and magnetic field irradiation has highly oriented α-from crystals. This study demonstrated that growth of this lamellar crystal is optimized when heated at 90 °C for 2 h. In addition, the effect of the ionic liquid can improve $X_{c}$ by maintaining lamella crystal orientation to some extent. Finally, in POM observation of the oriented PLLA film, it was revealed that the oriented lamellar crystals do not become spherulites due to their low isotropy.

## Figures and Tables

**Table 1:** *X*_c_ and crystal size of PLLA 10T films at each heat treatment time.

Sample Name	PLLA 10 T	PLLA 10 T2	PLLA 10 T4
*X*_c_	31	57	66
Crystallite size (nm)	14.86	22.95	24.64

## Supplementary Materials

The following are available online at http://www.mdpi.com/2073-4360/10/10/1083/s1, Figure S1: Relation between heat treatment time and *Xc* of no oriented PLLA films, Figure S2: Relation between heat treatment time and Xc of PLLAIL, Figure S3: XRD patterns of PLLAIL 0T films at each heat treatment time.
