# Supplementary material for: Preparation of Crystallites for Oriented Poly(Lactic Acid) Films Using a Casting Method under a Magnetic Field
Source: Polymers (Basel). 2018 Sep 29;10(10):1083. doi: 10.3390/polym10101083 (PMC6403967; doi:10.3390/polym10101083)
Supplement: Supplementary file 1 [file polymers-10-01083-s001.pdf]

Supporting Information for

Preparation of crystallites for orientied poly (lactic acid) films by casting method under magnetic field

Shuta Hara[1], Shuto Watanabe[1], Kohki Takahashi[2], Shigeru Shimizu[1] and Hiroki Ikake[1]

Contents

Fig.S1 Relation between heat treatment time and Xc of no oriented PLLA films.
Fig.S2 Relation between heat treatment time and Xc of PLLAIL
Fig.S3 XRD patterns of PLLAIL 0T films at each heat treatment time.

.

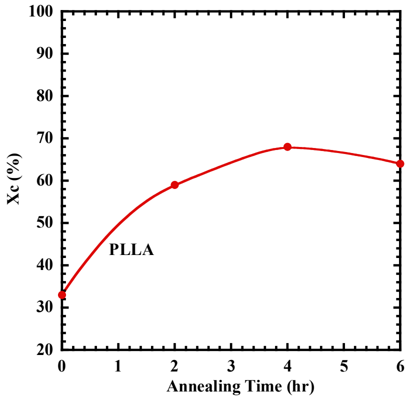

Fig.S1 Relation between heat treatment time and Xc of no oriented PLLA films.

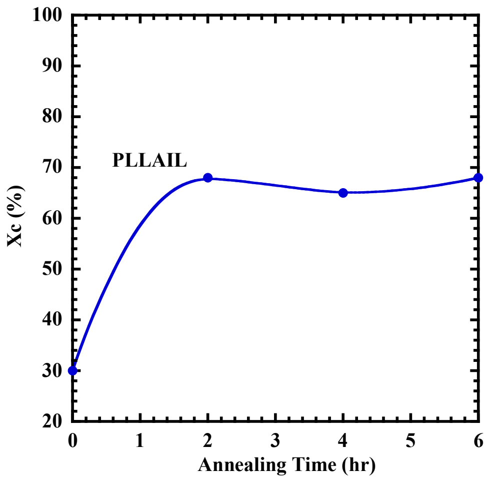

Fig.S2 Relation between heat treatment time and Xc of PLLAIL.

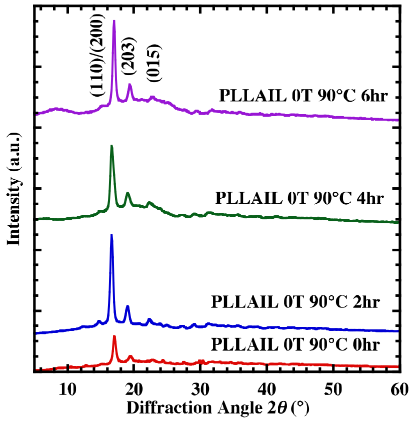

Fig.S3 XRD patterns of PLLAIL 0T films at each heat treatment time.
